# Comparative Analysis of MaxEnt and Deep Learning Approaches for Modeling Humpback Whale Distribution in North Iceland

**DOI:** 10.1002/ece3.71099

**Published:** 2025-03-18

**Authors:** Nils Barthel, Charla J. Basran, Marianne H. Rasmussen, Benjamin Burkhard

**Affiliations:** ^1^ Institute of Physical Geography and Landscape Ecology Leibniz University Hannover Hannover Germany; ^2^ Húsavík Research Center University of Iceland Húsavík Iceland

**Keywords:** comparative analysis, distribution models, environmental drivers, *Megaptera novaeangliae*, Skjálfandi Bay

## Abstract

In this study, we compared the established MaxEnt and a more novel deep learning approach for modeling the distribution of humpback whales ( *Megaptera novaeangliae* ) in north Iceland. We examined the mechanisms, structures, and optimization techniques of both approaches, highlighting their differences and similarities. Monthly distribution models for Skjálfandi Bay were created, from 2018 until 2021, using presence‐only sighting data and satellite remote sensing data. Search efforts and boat tracklines were utilized to create pseudo‐absence points for both models. Additionally, the trained models were used to create distribution projections for the year 2022, solely based on the available environmental data. We compared the results using the established area under the curve value. The findings indicate that both approaches have their limitations and advantages. MaxEnt does not allow continuous updating within a time series, yet it mitigates the risk of overfitting by employing the maximum entropy principle. The deep learning model is more likely to overfit, but the larger weight network increases the model's capability to capture complex relationships and patterns. Ultimately, the results show that the deep learning model had a higher predictive performance in modeling both current and future humpback whale distributions. Both modeling approaches have inherent limitations, such as the low resolution of the input data, spatial biases, and the inability to fully capture the entire complexity of natural processes. Despite this, deep learning showed promising results in modeling the distribution of humpback whales and prompts further research in different study areas and applications for other mobile animal species.

## Graphical abstract

This study compares the MaxEnt and deep learning approaches for modelling humpback whale distribution in north Iceland, highlighting their respective advantages and limitations. Overall, the findings indicate a higher predictive performance of the deep learning approach in modelling both current and future humpback whale distributions.

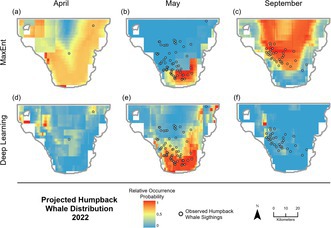

## 1 Introduction

Driven by underlying environmental influences, humpback whales ( *Megaptera novaeangliae* , hereafter HWs) undertake one of the largest annual migrations of any mammal, travelling between nutrient‐rich feeding grounds at high latitudes and warmer breeding grounds at lower latitudes. While the large‐scale distribution of HWs is well documented, their distribution at smaller spatial scales and the respective influence of environmental variables on this distribution remain less understood. Several environmental variables, such as temperature, prey availability, and bathymetry, are known to influence distribution patterns (Becker et al. 2017; Bamford et al. 2022; Stephenson et al. 2020). However, the degree to which these variables affect the habitat preferences of HWs at small spatial scales remains challenging to quantify (Meynecke et al. 2021).

Species distribution models (SDMs) are valuable tools for quantifying and visualizing the relationship between species and their environment. SDMs use environmental data and species occurrence records to determine the ecological niche of a species and derive its habitat preferences and spatial distribution. SDMs are particularly useful to fill in data gaps for species and areas where systematically collected field data may be scarce or difficult to obtain on a large spatial and temporal scale (Elith and Leathwick 2009). This makes SDMs a valuable resource in, for example, environmental planning and conservation efforts (Elith et al. 2011).

While traditional statistical modeling approaches such as generalized linear modelS (GLMs; Rockwood et al. 2020) and generalized additive models (GAMs; Becker et al. 2017) have been commonly used to develop SDMs, artificial intelligence (AI) approaches have become increasingly popular in recent years (Meynecke et al. 2021). This includes Boosted Regression Trees (BRT; Becker et al. 2020), Random Forest (Reisinger et al. 2021), and the Maximum Entropy (MaxEnt) approach (Smith and Santos 2020). The MaxEnt algorithm, developed by Phillips et al. (2004), was designed to address the challenge of working with presence‐only data, a common issue in species distribution modeling, by maximizing the entropy of its output. Since its development, MaxEnt has become one of the most widely used AI methods for modeling species distributions (Evangelista et al. 2011; Syfert et al. 2013) including HWs (Meynecke et al. 2021). For example, Smith et al. (2021) used MaxEnt to model HW distribution in the context of conservation planning near Australia, while Chou et al. (2020) examined the impact of anthropogenic activities on HW distribution off the West African coast. Other studies, such as Bombosch et al. (2014), applied MaxEnt to predict seasonal HW distributions in the Antarctic. Comparative studies by Derville et al. (2018), Fiedler et al. (2018), and Smith and Santos (2020) have demonstrated that MaxEnt is either comparable to or outperforms other modeling approaches, including GLMs, GAMs, and BRTs.

So far, more complex deep learning (DL) models have rarely been used and remain a relatively novel AI approach for creating SDMs, especially for HWs. A study by Purdon et al. (2020) applied a single‐layer artificial neural network, a model with just one hidden layer of neurons between the input and the output layer, to determine the distribution of HWs along the coast of southern Africa. The results showed lower predictive performance than the corresponding MaxEnt models. However, unlike single‐layer networks, DL models consist of multiple hidden layers, increasing their capacity to capture more complex, nonlinear relationships between species and environmental variables (Benkendorf and Hawkins 2020). Studies by Botella et al. (2018) and Deneu et al. (2021) demonstrated this improved capacity by modeling the distribution of a wide range of plant species in France using multi‐layer DL models. Similarly, Rew et al. (2021) applied DL to determine the distribution of different bird and reptile species in South Korea. Despite the slowly growing use of DL to determine SDMs, its application to cetaceans remains rare (Pasanisi et al. 2024). Recently, Cazau et al. (2023) applied DL to model the distribution of fin whales ( *Balaenoptera physalus* ) in the Mediterranean Sea. The study compared DL models to GAMs and found that the DL models achieved higher accuracy in estimating whale presence. The results of these studies showed that a DL model outperformed traditional AI models such as MaxEnt and BRTs or more conventional statistical models such as GAMs, indicating that the increased complexity of DL networks provides potential advantages over simpler models. Yet, given the relatively early stage of research comparing DL to other AI models such as MaxEnt, more case studies are needed to compare their predictive performances when modeling species distribution. Beyond this, to the best of our knowledge, no peer‐reviewed study has utilized DL to model the distribution of HWs.

This lack of using DL applications to model the distribution of HWs highlights the need for a comparative analysis between DL and the established MaxEnt approach. Therefore, this study aims to provide a comprehensive overview of the general functionality, strengths, and limitations of these two AI approaches by answering the following research questions: (1) What are the differences and similarities between MaxEnt and DL? (2) How do the results of the two modeling approaches differ? (3) Which approach is more suitable for modeling current and future HW distribution?

To achieve this, the functionality and implementation of both AI methods were described, followed by their application to create monthly SDMs of HWs in Skjálfandi Bay, North Iceland, covering the feeding season of March to October from 2018 to 2021. In a subsequent step, the trained models were applied to predict HW distribution from March to September 2022, based solely on the given environmental variables.

## 2 Methods

### 2.1 Study Area

**FIGURE 1:**
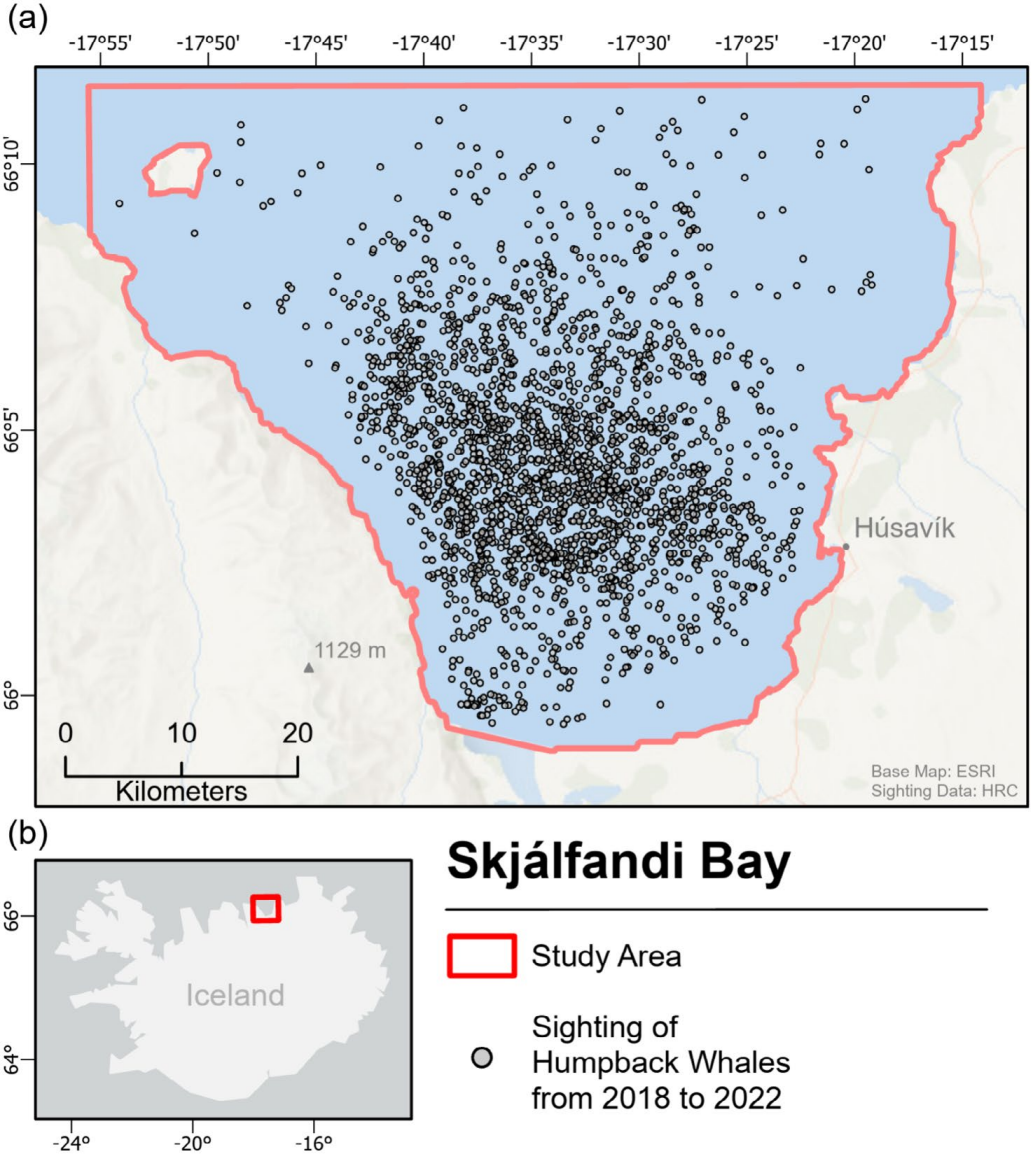
Map of (a) the study area in North Iceland, Skjálfandi Bay (red outline), including the location of humpback whale sightings from 2018 to 2022 used in this study (black circles) and (b) the location of the study area in Iceland (red box). Sighting data were provided by the University of Iceland's Research Center in Húsavík (HRC).

The study area, Skjálfandi Bay, is located on the northern coast of Iceland (66.1° N, 17.5° W) and covers approximately 550 km^2^ (Figure 1). The only town on the coastline of the bay is Húsavík, situated on the southeastern shore. This is where the University of Iceland's Research Center in Húsavík (HRC) is based and where whale‐watching surveys of the bay have their starting point. The melting snow from the western mountain range, the two freshwater rivers in the south, and the convergence of four large ocean currents (East Greenland, East Icelandic, North Atlantic, and the Icelandic Coastal Current) provide ideal conditions for nutrient‐rich water and the growth of phytoplankton (Lechwar et al. 2023). Due to its high productivity, the bay is a popular feeding ground for a range of cetaceans, including HWs. This, along with the availability of a large HW occurrence dataset, was the main reason for selecting this study area (Rasmussen 2009).

### 2.2 Data Collection and Pre‐Processing

#### 2.2.1 Humpback Whale Presence Points

The HW presence data were provided by the HRC, which collects the data onboard whale‐watching boat tours that cover large areas of the bay. The HRC works in cooperation with two whale‐watching companies, and at least two trained student researchers collect data on multiple tours per day, each lasting around 3 h, from March through November each year. The number of tours depends on the available daylight and weather conditions throughout the season, averaging 1.43 tours per day (based on trip data from 2018 to 2022). GPS location and search efforts are tracked throughout the tours. Researchers log a GPS sighting location at the beginning of a HW encounter. Subsequently, the HW's breathing rate and diving time are recorded during the encounter, and environmental data such as precipitation, cloud cover, visibility, and wind direction are noted every 30 min. Throughout the study period from 2018 to 2022, a total of 2540 HW sightings were recorded in the Skjálfandi Bay using this method. Of the available sightings, 1703 fell within the spatial extent of the available environmental data and were used in the modeling process (Table 1).

**TABLE 1:** Monthly number of humpback whale sightings and pseudo‐absence points, derived from search effort in hours and number of search days (Data provided by the University of Iceland's Research Center in Húsavík).

	March	April	May	June	July	August	September	October	Total
**2018**									
Sightings	2	8	97	82	97	92	26	34	438
Effort in hours	52	133	117	170	137	133	94	166	1002
Search days	10	27	30	29	29	26	22	23	196
Absence points	2580	644	618	825	694	663	4870	425	4614
**2019**									
Sightings	12	19	76	100	119	67	45	7	0445
Effort in hours	118	182	165	249	221	158	175	64	1332
Search days	25	29	25	29	28	23	26	19	180
Absence points	598	874	785	11,410	10,250	748	832	3560	6359
**2020**									
Sightings	13	10	15	60	47	73	37	43	298
Effort in hours	43	9	16	64	68	109	102	97	508
Search days	14	3	7	17	22	29	23	27	144
Absence points	245	051	103	341	381	580	523	5250	2749
**2021**									
Sightings	7	7	35	38	69	38	17	15	226
Effort in hours	42	55	165	81	192	179	98	34	846
Search days	10	18	26	19	29	25	17	11	165
Absence points	216	312	622	466	914	841	476	1900	4037
**2022**									
Sightings	8	2	55	85	89	25	32	—	296
Effort in hours	59	139	104	140	126	58	124	—	750
Search days	16	27	23	25	26	17	27	—	164
Absence points	315	693	529	685	634	319	633	—	3808

#### 2.2.2 Pseudo‐Absence Points

Modeling the distribution of a species typically requires both presence and absence data. However, datasets containing both presence and absence points, especially those collected systematically, are rare. In many cases, including this study, only presence data are available. Presence‐only data often come with an inherent spatial bias, which can affect the reliability and interpretability of the resulting distribution model (Phillips et al. 2009). This bias is caused by nonsystematic surveys, in which search effort is not uniform across the entire study area. For example, in this study, the search effort and the corresponding collection of presence data may be influenced by factors such as weather conditions, sea conditions, previous sightings, or proximity to the harbor.

To mitigate spatial bias in presence‐only data, pseudo‐absence points can be generated in a way that reflects the distribution of search effort, rather than being randomly assigned across the entire study area. One common method for generating pseudo‐absence points is the target group approach, described in detail by Phillips et al. (2009). However, if search effort data are available, it is generally preferred to use the data directly in the creation of pseudo‐absence points, as they provide a more accurate representation of sampling bias (Merow et al. 2013; Phillips et al. 2009).

For this study, both search efforts and boat tracklines were available. Following the method outlined by Derville et al. (2018), the number of pseudo‐absence points was determined by the total search effort in hours and the number of days on which at least one search was conducted:

(1) $$\text{Pseudo}‐\text{Absences}=\text{Effort}\left(h\right)\times 4+\text{Days}\times 5$$

**FIGURE 2:**
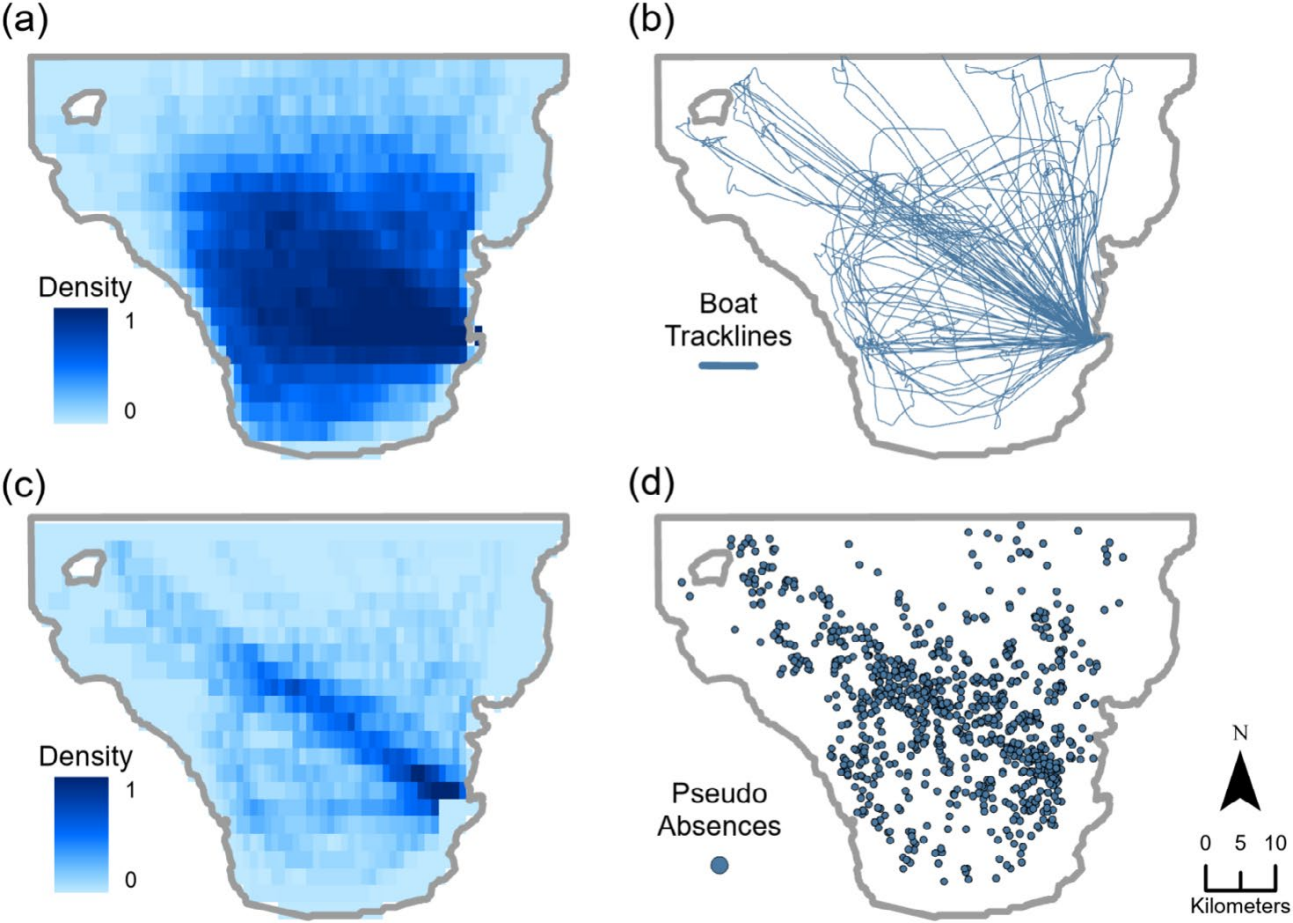
Overview of the pseudo‐absence point creation process based on boat trackline density. (a) Normalized boat trackline density across the full study period from 2018 to 2022. (b) Example visualization of boat tracklines recorded in April 2019, showing all tracklines across the study area. (c) The resulting density map is derived from the boat tracklines of April 2019. (d) Generation of pseudo‐absence points based on the April 2019 density map.

Thus, the number of pseudo‐absence points is directly proportional to the search effort (Table 1). The location of these points was then determined by the density of boat tracklines for each month (Figure 2). In this way, the spatial bias inherent in the presence‐only data could at least be partially balanced out (Fourcade et al. 2014).

#### 2.2.3 Environmental Variables

This study uses six environmental variables for the modeling process (Table 2). Sea surface temperature (SST), chlorophyll *a* concentration, and salinity at the sea surface vary over time; thus, a monthly mean was used. The other three variables, sea depth, seabed slope, and distance to shore, are constant.

**TABLE 2:** Environmental layers used in the modeling process, their resolution, and data source.

Layer	Description	Unit	Grid resolution (m)	Source and Technical information
SST	Sea surface temperature	°C	4638.3 × 4638.3	JAXA (2022); JAXA (2018), Kurihara (2020)
Chlorophyll *a*	Concentration in sea surface layer	mg/m^3^	4638.3 × 4638.3	JAXA (2022); JAXA (2018), Murakami (2020)
Salinity	Salinity in sea surface layer	PSU	8905.6 × 8905.6	NOPP (2022); Cummings and Smedstad (2013)
Depth	Depth based on a bathymetry chart	Meter	500 × 500	NOAA (2022); Amante and Eakins (2009)
Slope	Slope of the sea bedrock	Degree	500 × 500	Calculated with Slope tool in ArcGIS Pro
Distance to shore	Distance to the nearest shore	Meter	500 × 500	Calculated with Euclidean Distance tool in ArcGIS Pro

These six variables were selected based on data availability and their relevance in previous studies, which identified them as key influences on HW distribution (Bamford et al. 2022; Ersts and Rosenbaum 2003; Rosa et al. 2012; Smith et al. 2012; Stephenson et al. 2020; Purdon et al. 2020). For example, SST is a key indicator due to the temperature preferences of HWs and their prey. Chlorophyll *a* concentration serves as a proxy for prey availability, given the limited data on actual prey abundance. Salinity is influenced by cold, nutrient‐rich upwelling, which increases productivity and food availability for HWs, making it an important variable in their distribution (Meynecke et al. 2021; Rosa et al. 2012). Seabed slope can affect the upwelling of nutrient‐rich water, and HWs have been shown to prefer areas with specific depth ranges and proximity to the shore (Bamford et al. 2022; Smith et al. 2021).

The environmental data were obtained from Google Earth Engine, using the Global Change Observation Mission (GCOM) dataset (JAXA 2022) to derive SST and chlorophyll *a* concentration, along with salinity from the Hybrid Coordinate Ocean Model (HYCOM; NOPP 2022) and sea depth from the ETOPO Global Relief Model (NOAA 2022). The grid resolution ranged from 500 to 4638.6 m. The slope and distance to shore were calculated in *ArcGIS Pro 3.0* using the Slope and Euclidean Distance tools, respectively. Due to the absence of chlorophyll *a* data for October 2022, no SDMs were created for that specific month. Additionally, the depth data occasionally had values above sea level close to the shore due to the low spatial resolution of the data. Consequently, any values above sea level (≥ 0 m) were set to NA, so they did not influence the modeling process. Finally, all environmental data were resampled to a consistent grid resolution of 500 m.

The relationship between the environmental variables was first assessed using pairwise Pearson correlation, which revealed a strong negative linear correlation $\left(r=-0.87\right)$ between depth and distance to shore (Schober et al. 2018). To further assess the overall effect of the correlation between variables on the model's predictions, multicollinearity was tested by calculating the variance inflation factor (VIF). The VIF values for all variables were below 2, indicating low multicollinearity and that all variables still potentially provide unique information for the modeling process (Daoud 2017). Therefore, all variables were retained for the modeling process (Naimi and Araújo 2016; Purdon et al. 2020).

### 2.3 MaxEnt

MaxEnt, first introduced by Phillips et al. (2004), is a machine learning method employing the maximum entropy principle, a concept from information theory by Shannon (1948). The algorithm aims to determine a probability distribution that maximizes entropy and, thereby, minimizes unjustified assumptions based on the available information (Phillips et al. 2006). The main model inputs include occurrence data and environmental variables (covariates; Elith et al. 2011). To model nonlinear relationships, MaxEnt transforms the environmental variables into features using six different options: linear, product, quadratic, threshold, hinge, and category. Each environmental feature has an associated weight that determines the contribution of each feature to the final model (Merow et al. 2013).

The primary objective of the MaxEnt algorithm is to optimize the feature weights while satisfying two main constraints (Phillips et al. 2006). First, the environmental conditions of the final probability distribution and the known presence locations should have similar statistical properties, such as the same mean. Second, the final distribution should be as spread out as possible by incorporating the maximum entropy principle while still satisfying the first constraint. To determine the distribution with maximum entropy, MaxEnt utilizes a prior distribution based on pseudo‐absence points, commonly referred to as background points in the context of the MaxEnt algorithm. This prior distribution represents that with the highest possible entropy. MaxEnt aims to minimize the difference, that is, the relative entropy, between the estimated probability distribution and this prior distribution. Minimizing the relative entropy (Kullback–Leibler divergence) between the two distributions is the same as maximizing the entropy of the estimated probability distribution (Elith et al. 2011; Merow et al. 2013).

During the optimization process, all weights are initially set to 0 and are updated throughout multiple iterations. For this, MaxEnt aims to maximize a variation of the log‐likelihood function, which measures how well the prediction distinguishes between presence and pseudo‐absence points (Elith et al. 2011). In practice, this means that the model slowly diverges from the uniform distribution to determine the weights that best reflect the environmental conditions at presence points, satisfying the two constraints (Phillips et al. 2006).

To prevent overfitting, which occurs when the model fits too closely to the training data, an *L*_1_‐regularization term (LASSO) is added to the log‐likelihood function (Phillips et al. 2006). This results in the penalized log‐likelihood function also referred to as the gain function ($G\left(\eta \right)$):

(2) $$G\left(\eta \right)=\frac{1}{m}\sum_{i=1}^{m}\eta \left(x_{i}\right)-\log\sum_{i=1}^{n}Q\left(x_{i}\right)e^{\eta \left(x_{i}\right)}-L_{1}$$

where m is the number of presence points and n is the number of pseudo‐absence points (Merow et al. 2013). This process of optimization is repeated until the maximum log‐likelihood, that is, the maximum gain, or the user‐specified maximum number of iterations (the default is 500) is reached (Phillips 2017). The result is a relative probability ranging from 0 to 1, reflecting the preference or environmental niche of a species. The final probability distribution can also be described as a relative occurrence rate, sometimes referred to as an index for habitat suitability (Elith et al. 2011; Merow et al. 2013).

### 2.4 Deep Learning

Generally, an artificial neural network consists of multiple interconnected layers of neurons, including one input layer, one output layer, and one or more layers in between, referred to as hidden layers (Hinton et al. 2006). A neural network with multiple hidden layers is also referred to as a multi‐layer network or a deep learning network. Such deep learning networks have demonstrated better optimization and performance if compared to single neural networks (Botella et al. 2018). As additional hidden layers are added, the network can perform more complex calculations, enabling it to capture nonlinear relationships in the data. However, larger networks require more computational resources and are more prone to overfitting without proper regularization (Nielsen 2015).

A common neuron in a neural network consists of a set of weights, a bias term, and an activation function. The weights determine the importance of each input, and the bias allows the model to adjust the output. The weighted sum of the inputs is passed through the activation function, which is typically nonlinear and determines if and to what degree the information is passed on to the next layer of the network (Aggarwal 2018).

In a process called loss optimization, the weights and bias terms, starting with random values, are adjusted in each iteration using, in this case, the cross‐entropy loss function, which is considered to be very efficient for binary data such presence and absence (Nielsen 2015). This loss function (*L*) describes the difference between the predicted value $\left(\hat{y}\right)$ and the true value (*y*) and can be defined as follows:

(3) $$L\left(y,\hat{y}\right)=-\left(y\log\left(\hat{y}\right)+\left(1-y\right)\log\left(1-\hat{y}\right)\right)$$

The sum of the losses, calculated using Equation (3) across all data points, is referred to as the cost. The optimization, that is, training process, aims to minimize the cost by utilizing gradient descent and backpropagation (Aggarwal 2018; Nielsen 2015). To prevent the network from overfitting to the known data, a regularization function is typically applied. With this, by refining the model's weight parameters through multiple iterations, the network aims to capture the nonlinear relationships between environmental conditions and species presence or absence.

### 2.5 Model Development and Evaluation

The MaxEnt SDMs were created utilizing the MaxEnt software version 3.4.4 (Phillips et al. 2020). The hinge feature was chosen for feature transformation because it has demonstrated higher predictive performance for HW modeling (Bombosch et al. 2014; Derville et al. 2018). Additional settings, such as the strength of the *L*_1_‐regularization and the maximum number of iterations, were kept at their default values of 1 and 500, respectively.

**FIGURE 3:**
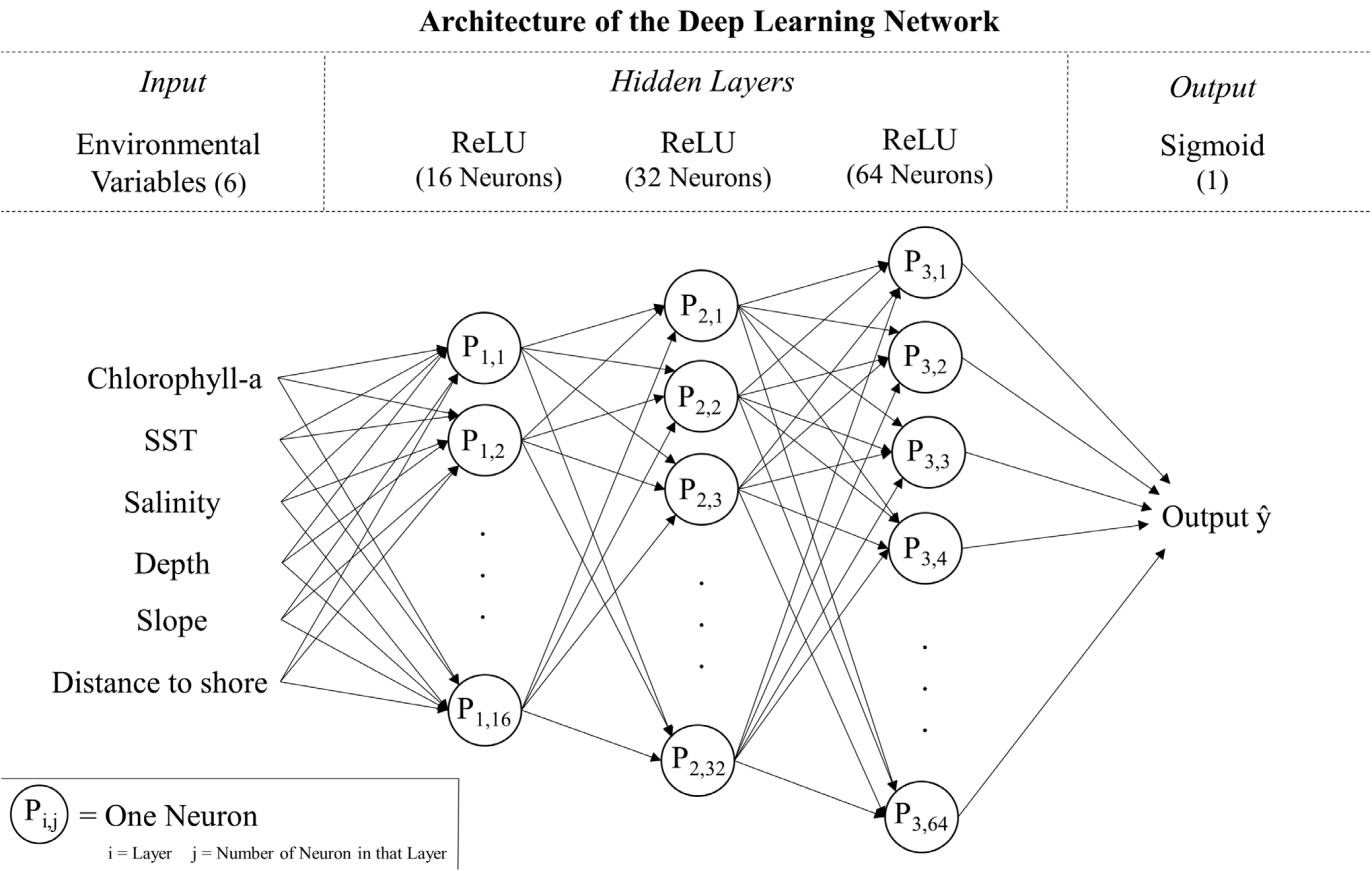
Visualization of the deep learning neural network architecture employed in this study, with six environmental variables as inputs, three hidden layers using the ReLU activation function, and an output layer utilizing the Sigmoid activation function.

The DL model was developed using Python and the TensorFlow Keras library. The architecture of the DL network in this study was a medium‐sized multi‐layer feedforward network with three hidden layers and an increasing number of neurons in each of the hidden layers: 16 in the first, 32 in the second, and 64 in the third (Figure 3).

The hidden layers in this specific neural network used the Rectified Linear Unit (ReLU) activation function, which offers a nonlinear transformation and is somewhat similar to the hinge function in the MaxEnt models (Botella et al. 2018). Furthermore, the ReLU activation function reduces the risk of the vanishing gradient problem, where the derivative of the loss function with respect to the weight parameters, and thus the gradient, becomes too small for effective learning (Nielsen 2015). The output layer utilized a sigmoid activation function to produce a probability distribution ranging from 0 to 1, indicating the likelihood of presence given the environmental conditions. During the training of the DL model, the same *L*_1_‐regularization term was used as in MaxEnt Equation (2) but with significantly lower strength due to the more complex structure of the neural network. This prevents overfitting while maintaining model optimization (Aggarwal 2018). The training process was continued until the cost function did not decrease for more than 5 iterations or a maximum of 500 iterations was reached.

MaxEnt and the DL network were applied for 32 months ranging from March 2018 to October 2021. This time frame was selected due to the limited availability of both environmental and sighting data from November to February. Unlike the DL model, the weights in a MaxEnt model must start at 0 each month, preventing updates from previous months. As a result, MaxEnt models were created from scratch each month, while DL models were continuously updated, utilizing the model of the previous month as a starting point for training each new model. Thus, the final DL model from October 2021 represents a continuously updated model based on data from 32 months, covering March 2018 to October 2021.

The results of both AI methods were evaluated using the established AUC value. A model with an AUC value < 0.6 is a model with bad quality, between 0.6 and 0.7 is considered a poor model, 0.7–0.8 is satisfactory, 0.8–0.9 is a good model, and above 0.9 is an excellent model with very high predictive abilities (Lissovsky and Dudov 2021; Merow et al. 2013). To improve the meaningfulness of the model performance evaluation a 10‐fold cross‐validation was applied. In this process, the dataset was split into 10 subsets, and for each fold, the model was trained on 9 subsets and evaluated on the remaining test subset, which was not used for training.

To assess the influence of each environmental variable on each monthly model, the permutation importance was determined. The permutation importance is calculated by randomly permuting each variable one at a time and measuring the resulting drop in the AUC value. A larger decrease in AUC indicates the greater importance of that variable in predicting the species distribution (Phillips 2017; Smith and Santos 2020).

Additionally, to assess how well both models could project HW distribution based solely on environmental data and previously trained models, seven projected SDMs were created for March to September 2022. The monthly MaxEnt projections were derived using a model based on sighting data and the average environmental conditions from the same month in previous years. For example, to predict May 2022, the average conditions from May 2018 to 2021 were used to train the model, assuming that the environmental conditions from previous years provide the best representation of future conditions for that month.

In contrast, the projection created with the DL algorithm was derived from the final continuously updated model, already trained and evaluated across the 32 months ranging from 2018 to 2022. Afterwards, to evaluate the projections, the AUC value was calculated by using the actual humpback whale sightings for the respective month as test data. The results provide insight into the generalizability of the models and how well they can handle unknown data, enabling an assessment of how suitable the models are for predicting future scenarios (Elith et al. 2010).

## 3 Results

### 3.1 Comparison Between MaxEnt and Deep Learning SDMs

The predictive performance of both models varied across the months and years. While the DL model generally outperformed MaxEnt, there were some months in which MaxEnt performed better. For example, in July of each year from 2018 to 2021, MaxEnt exhibited a slightly higher AUC than DL. Nevertheless, the monthly AUC of the DL was more consistently above 0.7. Out of the 32 SDMs, 23 MaxEnt models (72%) had an AUC above 0.7, while 30 DL models (94%) achieved this value and were at least satisfactory (Table 3). Overall, the average AUC for DL models was 0.801, compared to 0.753 for MaxEnt, indicating a 6% higher performance for the DL models across all months.

**TABLE 3:** AUC comparison of MaxEnt and deep learning (DL) SDMs from March 2019 to October 2021.

	March	April	May	June	July	August	September	October	Average
**2018**									
MaxEnt AUC	0.293	0.803	0.808	0.685	0.818	0.831	0.869	0.902	0.751
DL AUC	0.822	0.826	0.658	0.727	0.779	0.763	0.866	0.901	0.793
**2019**									
MaxEnt AUC	0.606	0.652	0.688	0.851	0.836	0.720	0.831	0.920	0.763
DL AUC	0.763	0.811	0.769	0.728	0.778	0.851	0.770	0.938	0.801
**2020**									
MaxEnt AUC	0.678	0.437	0.476	0.841	0.838	0.753	0.891	0.885	0.725
DL AUC	0.946	0.840	0.785	0.789	0.742	0.668	0.871	0.832	0.810
**2021**									
MaxEnt AUC	0.787	0.870	0.855	0.740	0.842	0.703	0.511	0.897	0.774
DL AUC	0.838	0.802	0.778	0.749	0.702	0.859	0.724	0.924	0.797

**FIGURE A1:**
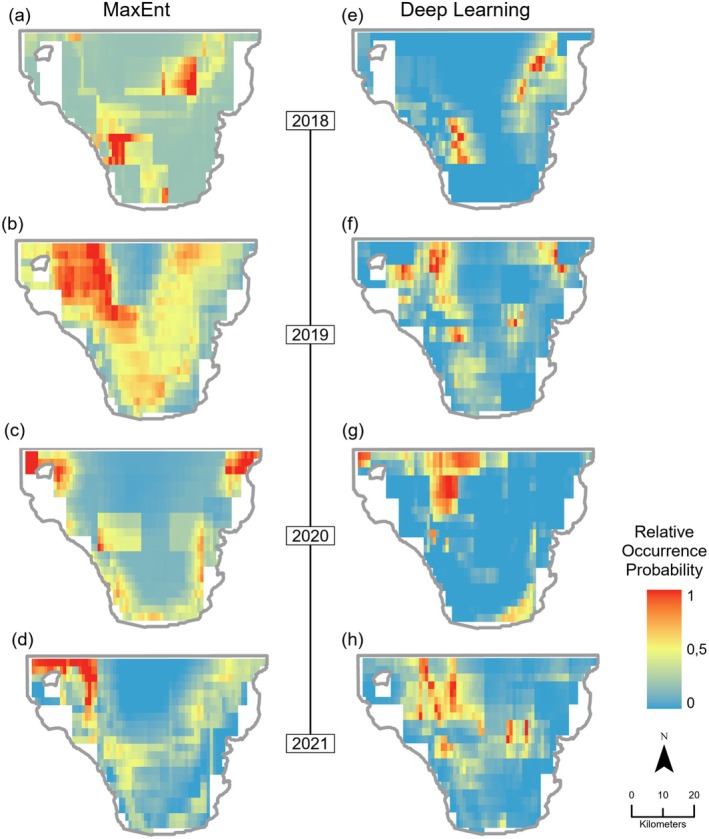
Comparison of humpback whale distribution models in March created with MaxEnt (a–d) and a deep learning model (e–h) in Skjálfandi Bay.

**FIGURE A2:**
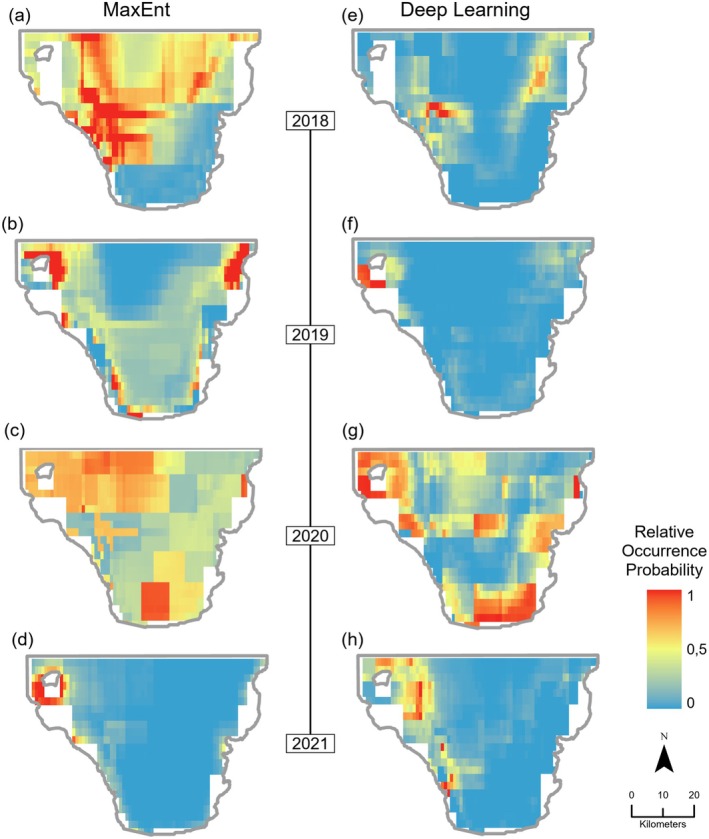
Comparison of humpback whale distribution models in April created with MaxEnt (a–d) and a deep learning model (e–h) in Skjálfandi Bay.

**FIGURE A3:**
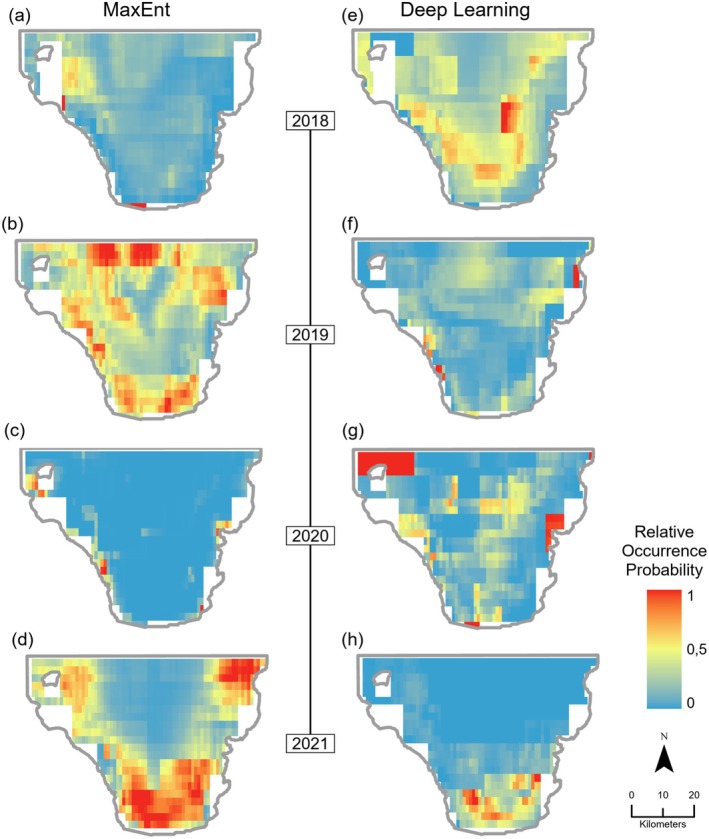
Comparison of humpback whale distribution models in May created with MaxEnt (a–d) and a deep learning model (e–h) in Skjálfandi Bay.

**FIGURE A4:**
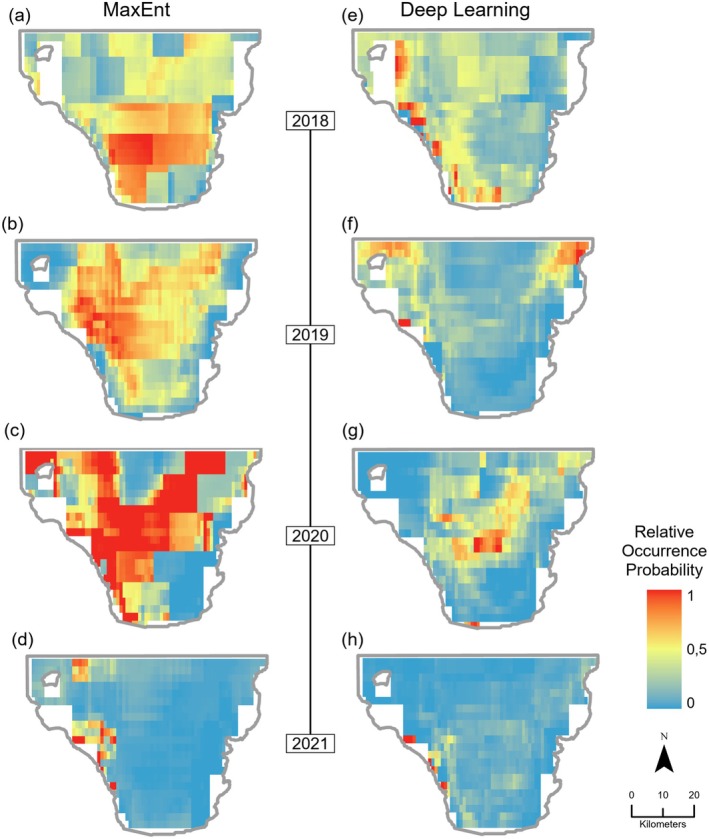
Comparison of humpback whale distribution models in June created with MaxEnt (a–d) and a deep learning model (e–h) in Skjálfandi Bay.

**FIGURE A5:**
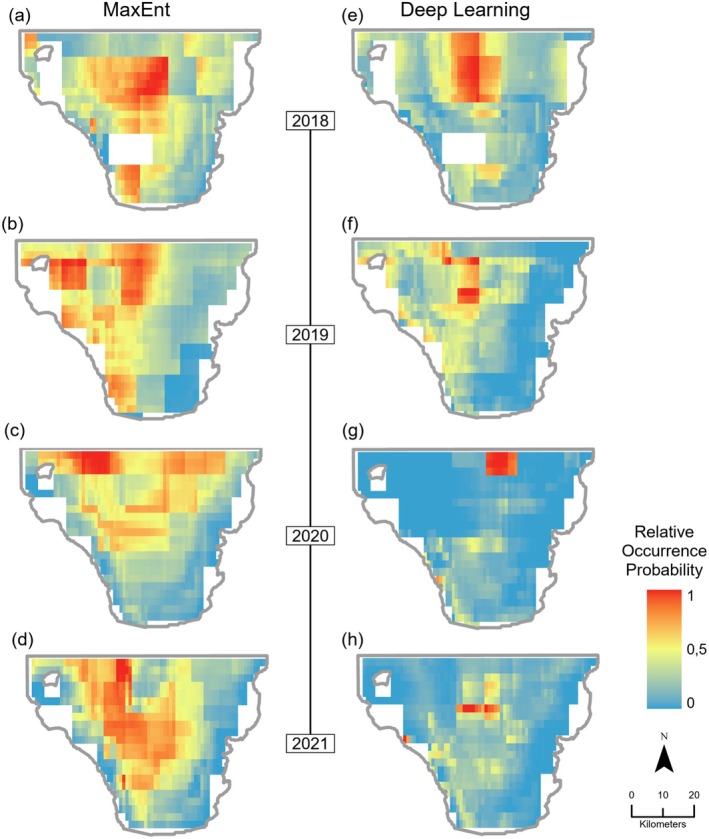
Comparison of humpback whale distribution models in July created with MaxEnt (a–d) and a deep learning model (e–h) in Skjálfandi Bay.

**FIGURE A6:**
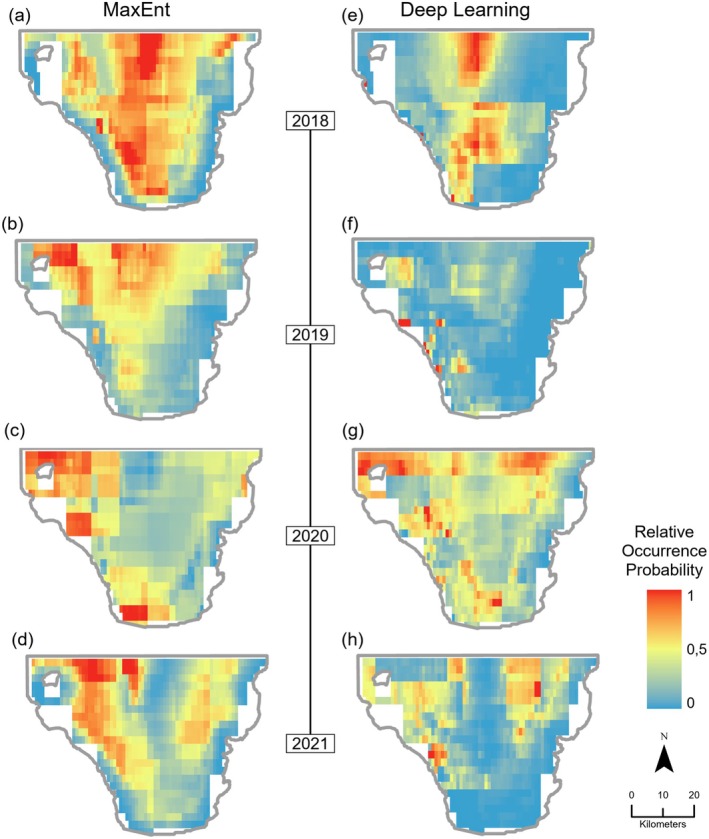
Comparison of humpback whale distribution models in August created with MaxEnt (a–d) and a deep learning model (e–h) in Skjálfandi Bay.

**FIGURE A7:**
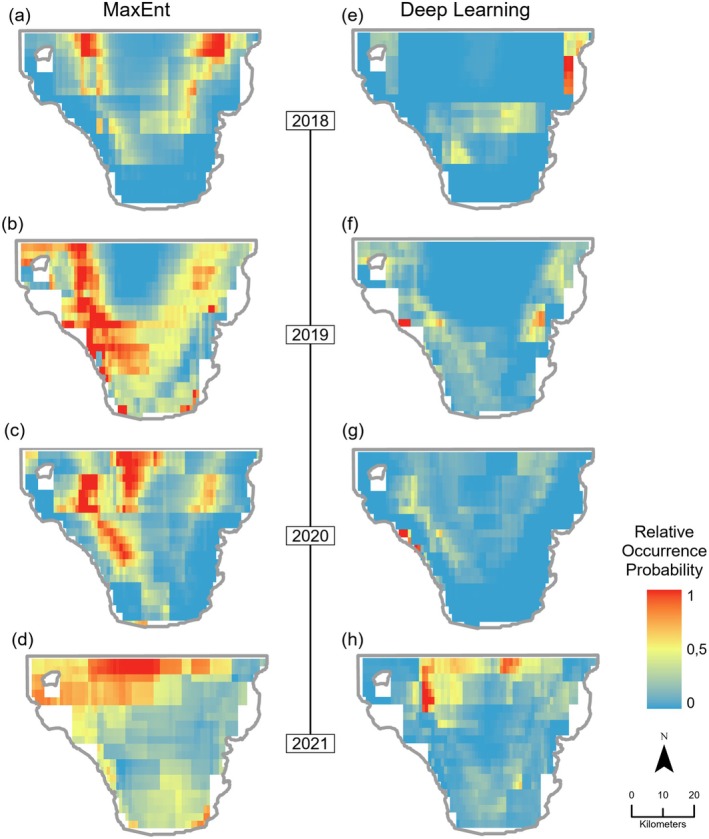
Comparison of humpback whale distribution models in September created with MaxEnt (a–d) and a deep learning model (e–h) in Skjálfandi Bay.

**FIGURE A8:**
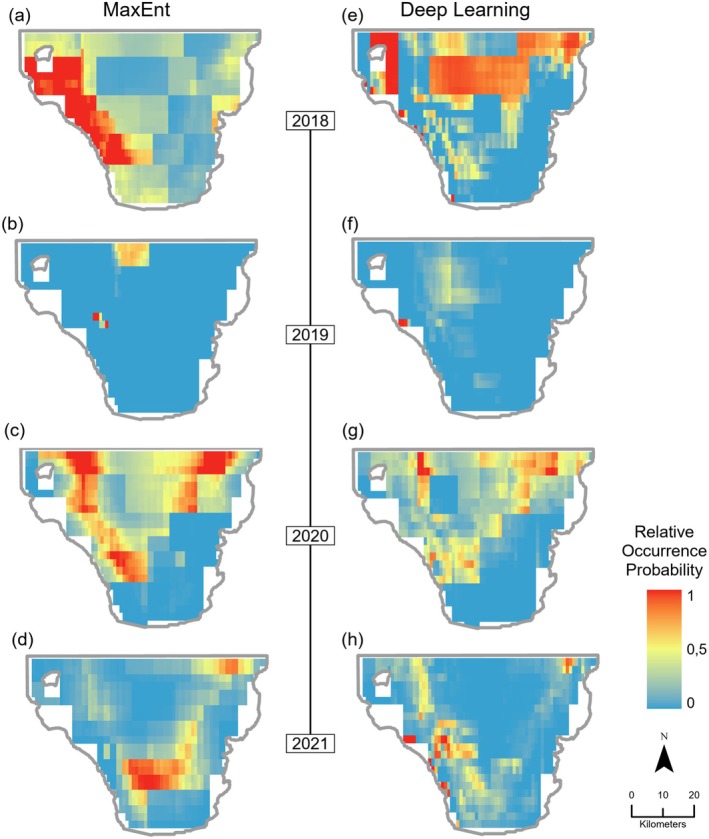
Comparison of humpback whale distribution models in October created with MaxEnt (a–d) and a deep learning model (e–h) in Skjálfandi Bay.

Although there were occasional similarities in the probability distribution within the same month across years, the modeled distribution showed no clear monthly or seasonal pattern (Appendix 1: Figures A1, A2, A3, A4, A5, A6, A7, A8). Similarly, there was no consistent trend in the calculated permutation importance (Table A1), as each monthly SDM relied on different variables. For example, in September 2018, the MaxEnt model relied heavily on the variable depth, with a calculated importance of 71.8%, while in the DL model, its importance was only 0.6% for the same month. On average, over the 4 years, the DL models exhibited a more even distribution of importance across all variables, with values ranging from 14.4% for SST to 21.1% for distance to shore (Table 4). In contrast, the MaxEnt model showed a greater variation, with average permutation importance ranging from 8.6% for slope to 26.1% for salinity. Despite this, the standard deviation for each variable across all months was high in both models, though the DL model showed slightly less variability compared to MaxEnt.

**TABLE 4:** Average and standard deviation (SD) of permutation importance (%) for MaxEnt and deep learning models across the six environmental variables sea surface temperature (SST), chlorophyll *a* concentration, salinity, depth, slope, and distance to shore.

Variable	MaxEnt		Deep learning	
	Average	SD	Average	SD
SST	12.9	10.7	14.4	7.3
Chlorophyll *a*	18.4	13.6	17.2	9.8
Salinity	26.1	16.6	17.3	9.0
Depth	20.6	15.8	14.6	10.6
Slope	8.6	8.5	16.3	8.8
Distance to shore	13.5	10.7	21.1	11.3

### 3.2 Projections of SDMs

**FIGURE 4:**
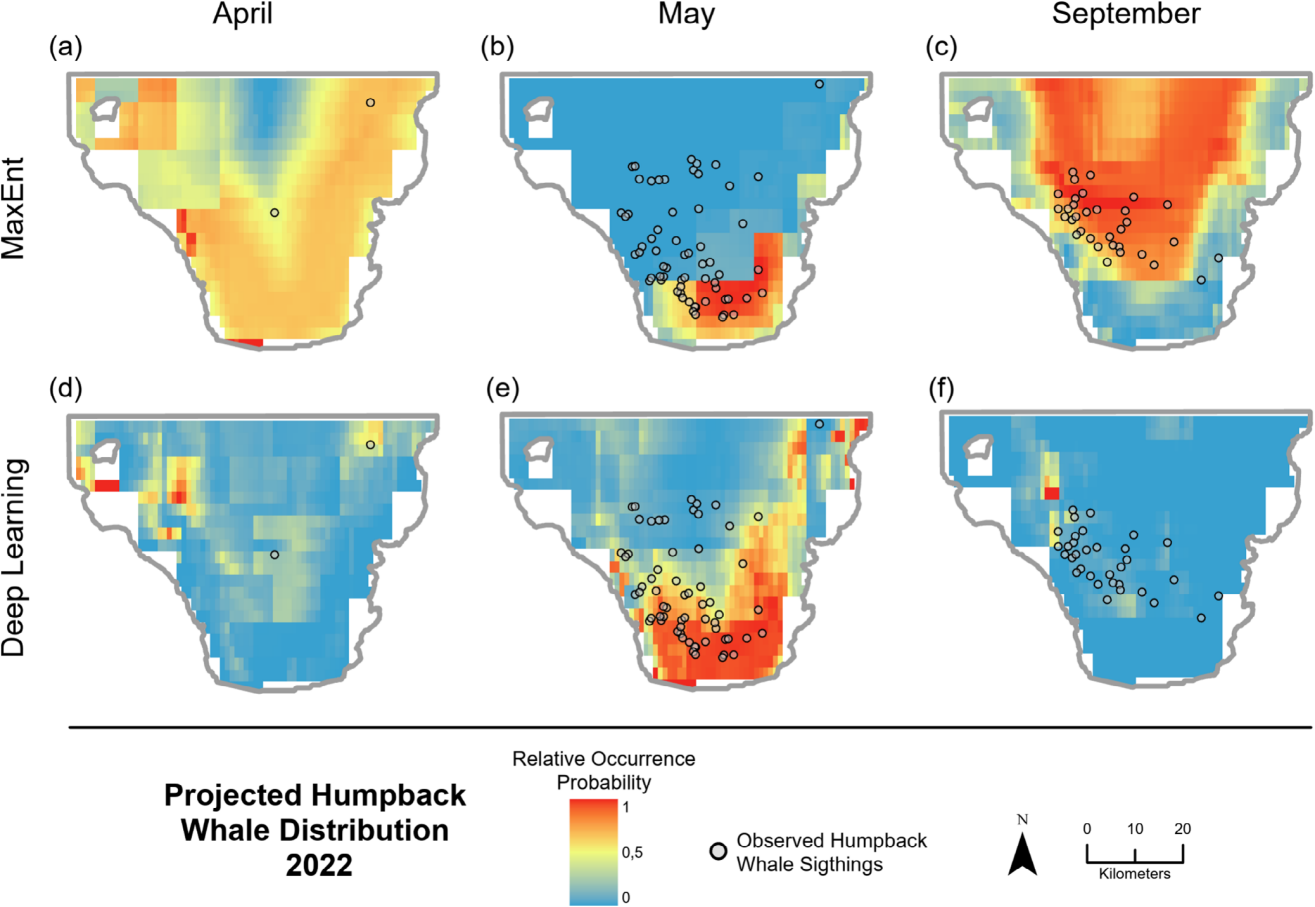
Visualization of projected humpback whale distribution for 2022 created with MaxEnt (a–c) and the Deep Learning model (d–f) in the months where the deep learning model achieved an AUC value over 0.7. Black circles represent the actual recorded HW sightings.

MaxEnt and the DL network were also utilized to create projected SDMs, based on trained models and environmental data for 2022. In three out of the seven modeled months, the DL model was able to somewhat accurately model the distribution of HWs in 2022 (Figure 4). On the other hand, all MaxEnt projections had an AUC value below 0.7 (Table 5). The highest AUC value of 0.851 was achieved in the projected DL model for April 2022. However, the AUC value was based on only two presence points. The significance of the respective AUC value is very low and, therefore, does not provide an accurate assessment of the predictive performance.

**TABLE 5:** AUC values for projected MaxEnt and deep learning (DL) SDMs for 2022, with monthly AUC values from March to September and average AUC for the year.

	March	April	May	June	July	August	September	Average
MaxEnt AUC	0.651	0.474	0.524	0.647	0.575	0.610	0.613	0.585
DL AUC	0.415	0.851	0.704	0.549	0.539	0.623	0.764	0.635
Sightings	8	2	55	85	89	25	32	

Nevertheless, there were enough presence points for May and September for a better assessment. In both months, the DL model had satisfactory predictive performance, with an AUC of 0.704 in May and 0.764 in September. Overall, the predicted distributions of HWs produced by the two methods, MaxEnt and DL, were very different. While the MaxEnt model assigned a high relative occurrence probability to a large area of the bay in both April and September, the DL model was less spread out in both months, showing only small areas of high relative probability (Figure 4). This was slightly different in May, where both models identified a high probability in the southern part of the bay. However, the DL model also predicted a higher relative occurrence probability in the eastern area of the bay, resulting in higher accuracy.

## 4 Discussion

### 4.1 Model Comparison

The key findings of this study are structured around answering the three research questions regarding the differences and similarities between both methods, comparing the results, and evaluating their suitability for modeling HW distributions. The DL network was specifically designed to be comparable to the MaxEnt algorithm, and generally, the application of both methods resulted in similar AUC values for some of the 32 modeled months and somewhat similar spatial patterns. Nonetheless, there are still some notable differences in the underlying mechanisms of both methods, affecting the resulting SDMs and their interpretation.

One main difference is the optimization process of both methods. MaxEnt utilizes the pseudo‐absences, that you, background points, and a penalized log‐likelihood function (Equation 2) to gradually diverge from a uniform distribution, maximizing the entropy. This process ensures that MaxEnt models often generalize well and, as can be seen in the results of this study, produce dispersed distribution models with high predictive performance in most months.

In contrast, DL uses a cross‐entropy loss function and treats pseudo‐absences as true absences, which increases the potential for overfitting, especially with small datasets. Yet, in this study, the DL model also generalized well, benefiting from the same $L_{1}$ regularization as the MaxEnt algorithm. Almost all 32 modeled SDMs achieved at least a satisfactory AUC value and outperformed MaxEnt during most months. Furthermore, the results suggest that the DL model is better suited for modeling future predictions. However, it is important to note that, since the DL model only achieved an AUC value above 0.7 in two out of the seven projected months, it cannot be confidently claimed that either trained model is fully reliable in predicting future distributions in this study. This is likely due to the short timeframe and the limitations of the available data.

Two main factors may explain why the DL model outperformed MaxEnt in this study and why it is potentially better suited for modeling both current and, at least partially, future HW distributions. First, the DL model does not require its weight parameters to start at zero, allowing it to be continuously updated across months. This capability enables the DL model to indirectly utilize the entire dataset, capturing underlying patterns over time. Second, unlike MaxEnt, the DL model consists of a large weight network with multiple layers, allowing it to capture more complex, nonlinear relationships between the input variables and the relative probability of humpback whale occurrence. However, it is important to note that this increased complexity comes with higher computational costs, particularly when the dataset is much larger than the one used here.

The results support the findings of other studies that applied DL models to different species, such as Botella et al. (2018) and Rew et al. (2021), where DL models outperformed the established MaxEnt approach. Additionally, the results also align with the study by Cazau et al. (2023), which demonstrated the potential of a DL model to predict the distribution of cetaceans.

### 4.2 Importance of the Environmental Variables

Overall, all selected environmental variables were important in determining the distribution of HW in the bay, supporting previous studies that highlight the relevance of these variables (e.g., Ersts and Rosenbaum 2003; Smith et al. 2012; Stephenson et al. 2020; Purdon et al. 2020). However, the importance varied strongly depending on the month and the model. Similar to the modeled spatial distribution, there was no distinct monthly or seasonal pattern describing the relationship between the environmental variables and the relative occurrence probability.

Although depth and distance to shore had a high negative pairwise correlation, the monthly permutation importance indicates that neither variable was redundant for the modeling process. Both variables provided unique information, with one sometimes being more important than the other, while in other months, both showed similar levels of importance.

### 4.3 Limitations

The two methods, as well as the modeling approach in general, have various limitations. Apart from the inherent limitations of the algorithms discussed earlier, the input data and the overall modeling approach also imposed limitations on the results. One key limitation often encountered in SDMs is the sampling bias of the presence data (Fiedler et al. 2018). The data were not systematically collected, covered only a relatively small study area, and captured only a momentary snapshot of a highly mobile species. Although the spatial bias was addressed by adopting the approach of Derville et al. (2018), the spatial bias could not be fully eliminated or replaced by systematic surveys that provide true absence data (Syfert et al. 2013). The quantity of presence points can be another limiting factor for model creation and validation. A very low number of presence points affects the model's capability to detect patterns in the data (Becker et al. 2010).

Environmental data in SDMs are often derived from satellite remote sensing datasets (e.g., Becker et al. (2010), Rosa et al. (2012), Smith et al. (2012), Purdon et al. (2020)). Similarly, this study relies on satellite data for environmental variables, including SST, chlorophyll *a* (grid resolution of 4638 m), and salinity (grid resolution of 8906 m). However, these datasets introduce limitations due to their relatively low spatial resolution and use of monthly averages. The data were used to reflect environmental conditions that are temporally and spatially highly variable. Consequently, they do not necessarily capture the real environmental conditions at the specific location and time when the presence point was recorded. In addition, satellite remote sensing data are highly influenced by the limitations of the corresponding sensor of the satellite and the specific processing algorithms of the data (McPherson et al. 2006).

The analysis of the permutation importance (Table 4) highlights some difficulties in modeling HW distribution and determining key environmental influences. The high standard deviation shows that there are no definitive environmental factors for predicting HW distribution in the bay across all months. There are several possible explanations for this, including the mentioned low spatial and temporal resolutions of the input data and the influence of variables that were not included in this study, such as nearby boats or social interactions among whales (Chou et al. 2020; Smith et al. 2021). The SDMs in this study are only stochastic approximations based on a limited number of variables, monthly averages, and momentary locations of HWs. Therefore, the derived SDMs can depict the complex and dynamic natural processes only to a certain degree (Pasanisi et al. 2024). Thus, the high standard deviation of the average permutation importance does not necessarily contradict the validity of the model itself, but could be caused by the complex interactions of natural processes.

### 4.4 Future Research

The results of this study provide insights into the differences between the two AI approaches, highlighting the potential and limitations of novel distribution modeling methods like DL. However, using DL for SDM, especially for HWs, is still a novel approach and requires further extensive research. The limitations of the input data could be dealt with by comparing study results with GPS‐tracking data or high‐resolution satellite imagery (Cubaynes et al. 2018; Meynecke and Liebsch 2021; Stanistreet et al. 2013). Additionally, it could offer new insights to compare the results with other satellite and drone remote‐sensing data or to explore the possibilities of implementing in situ measurements.

The comparison of models showed superior performance for the DL model, but further testing is needed for different study areas, timeframes, and species (Pasanisi et al. 2024). An extended study conducted over a longer timeframe and a larger area may further validate the results, enable a better assessment of possible patterns over time, and provide additional insights into the performance of the DL model. A DL model based on an extended timeframe with sufficient predictive performance can then be employed to assess the influence of various scenarios, such as the effects of climate change on HW distribution.

Many new potential research opportunities arise through modifications of the DL neural network by utilizing different architectures. This could include a Convolutional Neural Network (CNN; e.g. Chauhan et al. 2018) or specialized Recurrent Neural Networks (RNN) utilizing Long Short‐Term Memory (LSTM) units to further address the vanishing gradient problem and detect long‐term patterns (Hochreiter and Schmidhuber 1997). Other, more recent examples of neural network architectures include the implementation of so‐called Transformers (Irie et al. 2019) or Liquid Neural Networks (Chahine et al. 2023). Applying such different neural network architectures to model SDMs and comparing the results makes it possible to draw conclusions about the suitability of these architectures. This could provide a more comprehensive understanding of utilizing DL to model the distribution of a species, including its respective limitations, advantages, and possibilities.

## 5 Conclusion

This study highlights the key aspects of MaxEnt and DL for modeling HW distribution in north Iceland, identifying differences, similarities, and limitations. Despite the mentioned limitations, both approaches were able to accurately model HW distribution, achieving at least satisfactory accuracies in most months. However, DL often outperformed MaxEnt while modeling current and future distributions. The DL network offers a range of possibilities and has some key advantages compared to the more established MaxEnt approach. The flexible and complex structure of a DL neural network is the reason why neural networks are well suited for detecting complex relationships, though it can potentially lead to overfitting. Additionally, a DL model can be updated throughout a time series and therefore, in theory, is better suited for detecting long‐term patterns.

The results of this study show promise for the use of DL and its potential to provide deeper insights into the various influences on species distribution and enhance the accuracy of SDMs. Given this, future testing should involve larger datasets, higher‐resolution environmental data, and applying DL with different neural network architectures to model the distribution of other species across larger study areas to refine this approach.

## Author Contributions

**Nils Barthel:** conceptualization (lead), methodology (lead), software (lead), visualization (lead), writing – original draft (lead), writing – review and editing (equal). **Charla J. Basran:** conceptualization (supporting), writing – original draft (supporting), writing – review and editing (equal). **Marianne H. Rasmussen:** conceptualization (supporting), writing – review and editing (equal). **Benjamin Burkhard:** conceptualization (supporting), methodology (supporting), supervision (lead), writing – original draft (supporting), writing – review and editing (equal).

## Conflicts of Interest

The authors declare no conflicts of interest.

## Data Availability

The data and code used in this study are publicly available in the Zenodo repository and can be accessed via the DOI: 10.5281/zenodo.13167799.

## Appendix 1

### Monthly Permutation Importance

**TABLE A1:** Monthly permutation importance of depth, distance to shore (Distance), slope, chlorophyll *a* (Chl. *a*), sea surface temperature (SST), and salinity using MaxEnt and deep learning models for the years 2018–2021.

Year	Month	MaxEnt						Deep learning					
		Depth	Distance	Slope	Chl. *a*	SST	Salinity	Depth	Distance	Slope	Chl. *a*	SST	Salinity
2018	March	5.6	50.0	0.0	44.4	0.0	0.0	4.9	68.2	0.0	10.8	11.4	11.9
	April	13.7	4.5	42.7	24.3	0.0	14.8	2.3	24.9	16.7	32.3	10.8	12.9
	May	7.0	5.9	9.6	0.1	10.1	67.3	33.5	17.2	0.0	10.1	20.8	21.9
	June	6.9	12.9	1.3	8.3	31.9	38.7	3.1	4.1	19.2	19.1	30.9	23.6
	July	4.2	30.1	0.6	51.0	13.5	0.6	0.0	27.7	20.1	5.9	7.3	39.0
	August	4.5	26.3	1.9	48.6	16.0	2.8	4.5	39.0	8.6	14.2	15.0	18.7
	September	71.8	3.6	1.7	12.4	2.2	8.2	0.6	11.4	23.9	13.8	28.0	22.3
	October	18.0	11.9	0.9	44.5	9.8	15.0	18.0	25.9	33.3	5.5	7.7	9.7
	Average	16.5	18.1	7.3	29.2	10.4	18.4	8.4	27.3	15.2	14.0	16.5	20.0
	SD	14.2	13.0	9.4	17.9	7.5	17.3	8.7	13.3	9.3	5.9	7.6	6.7
2019	March	63.7	11.6	0.7	22.5	0.6	0.8	38.6	13.0	26.2	10.6	8.7	2.8
	April	51.9	2.8	0.9	37.0	3.6	3.9	11.6	8.3	18.1	27.5	8.2	26.3
	May	3.6	25.7	1.6	26.6	7.0	35.5	1.0	15.1	29.7	3.0	19.6	31.7
	June	9.8	27.8	5.3	1.9	8.0	47.3	23.7	20.7	32.1	4.8	10.3	8.3
	July	11.1	2.1	1.4	10.5	25.1	49.8	21.2	16.3	7.2	33.2	11.7	10.4
	August	63.8	7.6	2.1	4.3	2.0	20.1	19.4	29.6	8.1	16.1	17.9	8.9
	September	18.1	6.9	17.9	38.2	12.3	6.6	4.0	16.6	55.5	2.9	11.2	9.8
	October	11.5	0.0	2.9	33.2	0.1	52.2	18.2	21.3	0.0	44.2	8.3	8.0
	Average	29.2	10.6	4.1	21.8	7.4	27.0	17.2	17.6	22.1	17.8	12.0	13.3
	SD	23.0	8.4	3.7	12.2	5.8	19.2	8.8	4.7	13.8	12.9	3.4	7.9
2020	March	21.7	1.7	7.8	5.4	53.0	10.4	4.4	39.6	10.1	7.4	10.8	27.8
	April	0.4	6.7	25.8	2.4	43.9	20.9	13.8	0.9	12.4	51.2	0.0	24.4
	May	11.0	5.1	25.3	7.7	0.0	50.9	5.4	26.4	16.7	44.5	0.2	6.8
	June	6.8	6.4	18.1	29.1	12.2	27.4	24.1	9.9	13.7	11.2	32.3	8.9
	July	12.5	2.6	1.6	0.0	1.6	81.6	9.3	3.0	10.5	22.8	33.8	20.5
	August	13.7	23.1	3.5	21.4	24.1	14.1	1.9	33.1	8.2	16.5	26.5	13.9
	September	6.8	31.5	30.2	1.1	4.6	25.9	67.3	28.4	0.7	11.3	0.0	0.0
	October	0.9	14.8	7.0	29.3	24.9	22.9	17.7	18.9	17.7	8.3	13.7	23.7
	Average	9.2	11.5	14.9	12.1	20.5	31.8	18.0	20.0	11.2	21.6	14.7	15.8
	SD	5.5	8.7	9.9	10.9	15.9	17.2	13.9	11.8	3.9	13.4	12.1	8.4
2021	March	53.3	6.1	0.7	11.4	0.2	28.3	30.1	2.7	13.7	27.3	10.9	15.4
	April	28.5	0.6	13.6	17.6	0.7	39.1	32.6	4.8	8.1	15.9	11.8	26.7
	May	33.7	4.5	4.9	3.1	28.4	25.4	6.7	31.1	35.2	5.2	24.8	0.0
	June	20.6	11.5	5.4	1.6	16.2	44.7	12.4	30.7	14.6	12.8	13.2	16.4
	July	7.7	10.8	4.1	24.7	11.3	41.4	11.9	50.7	18.0	14.0	0.0	10.7
	August	22.1	49.1	2.7	16.9	4.6	4.7	15.7	19.0	9.2	15.1	21.8	19.3
	September	5.0	26.9	30.1	5.8	10.0	22.2	5.0	1.1	12.2	5.1	16.0	60.6
	October	48.1	0.0	1.4	2.4	36.2	11.9	5.3	16.8	22.8	26.5	15.9	12.6
	Average	27.4	13.7	7.9	10.4	13.5	27.2	15.0	19.6	16.7	15.2	14.3	20.2
	SD	13.5	12.2	7.0	7.2	10.1	11.2	8.4	13.4	6.5	6.0	5.3	11.7
Overall average		20.6	13.5	8.6	18.4	12.9	26.1	14.6	21.1	16.3	17.2	14.4	17.3
Overall SD		15.8	10.7	8.5	13.6	10.7	16.6	10.6	11.3	8.8	9.8	7.3	9.0

### Visualization and Comparison Between MaxEnt and Deep Learning SDMs

The following figures depict the monthly distribution of humpback whales from 2018 to 2021 created with MaxEnt and Deep Learning.
